# Comparison of *in vivo* and *in vitro* models to evaluate pulp temperature rise during exposure to a Polywave^®^ LED light curing unit

**DOI:** 10.1590/1678-7757-2018-0480

**Published:** 2019-05-20

**Authors:** Patricio Runnacles, Cesar Augusto Galvão Arrais, Cristiane Maucoski, Ulisses Coelho, Mario Fernando De Goes, Frederick Allen Rueggeberg

**Affiliations:** 1 Universidade Estadual de Ponta Grossa, Departamento de Odontologia Restauradora, Ponta Grossa, Paraná, Brasil.; 2 Universidade Estadual de Campinas, Faculdade de Odontologia de Piracicaba, Departamento de Materiais Dentários, Piracicaba, São Paulo, Brasil.; 3 Augusta University, Dental College of Georgia, Department of Restorative Sciences, Dental Materials Section, Augusta, Georgia, USA.

**Keywords:** Bicuspids, Volunteers, Dental curing lights, Temperature

## Abstract

**Objectives:**

To measure and compare *in vivo* and *in vitro* pulp temperature (PT) increase (ΔTEMP) over baseline, physiologic temperature using the same intact upper premolars exposed to the same Polywave^®^ LED curing light.

**Methodology:**

After local Ethics Committee approval (#255,945), local anesthesia, rubber dam isolation, small occlusal preparations/minute pulp exposure (n=15) were performed in teeth requiring extraction for orthodontic reasons. A sterile probe of a temperature measurement system (Temperature Data Acquisition, Physitemp) was placed within the pulp chamber and the buccal surface was sequentially exposed to a LED LCU (Bluephase 20i, Ivoclar Vivadent) using the following exposure modes: 10-s low or high, 5-s Turbo, and 60-s high. Afterwards, the teeth were extracted and K-type thermocouples were placed within the pulp chamber through the original access. The teeth were attached to an assembly simulating the *in vivo* environment, being similarly exposed while real-time temperature (°C) was recorded. ΔTEMP values and time for temperature to reach maximum (ΔTIME) were subjected to two-way ANOVA and Bonferroni's *post-hoc* tests (pre-set alpha 0.05).

**Results:**

Higher ΔTEMP was observed *in vitro* than *in vivo*. No significant difference in ΔTIME was observed between test conditions. A significant, positive relationship was observed between radiant exposure and ΔTEMP for both conditions (*in vivo*: r2=0.917; p<0.001; *in vitro*: r2=0.919; p<0.001).

**Conclusion:**

Although the *in vitro* model overestimated *in vivo* PT increase, *in vitro* PT rise was close to *in vivo* values for clinically relevant exposure modes.

## Introduction

Maintenance of pulp safety is an essential challenge for clinicians in many restorative treatments, since heat generated from use of high and low speed handpieces,^1^ restorative materials having exothermic setting reactions,^2^ restoration finishing and polishing,^3^ as well as from application of high power light emitting diode (LED) - based light curing units (LCUs) and laser sources to polymerize resin-based materials^4^ may cause pulp temperature (PT) to rise to values considered harmful for the pulp.^5^ For these reasons, *in vitro* temperature increase within pulp chamber of extracted teeth has been investigated.^4^ ^,^ ^6^ Over the last decade, heat generated during tooth exposure to light emitted by LED LCUs has become an area of concern for clinicians and researchers. These concerns are based on availability of new, powerful light-curing devices that are capable of emitting light with radiant emittance values exceeding 2,000 mW/cm^2^.^7^

Several studies evaluated the thermal stimulus caused by LED LCUs. Most of those studies relied on *in vitro* techniques using extracted teeth to evaluate temperature rise within the pulp chamber of extracted teeth while external heat sources were applied.^6^ ^,^ ^8^ ^,^ ^9^ The most common methodology uses thermocouples inserted inside pulp chambers of extracted teeth to measure temperature changes in this location during exposure to various LCUs.^6^ ^,^ ^8^ ^,^ ^10^ ^,^ ^11^ In an attempt to simulate the same physiological conditions observed *in vivo*, some authors developed specific devices in which the roots of extracted teeth were connected to a pump to provide a water fluid flow inside the pulp chamber so blood flow could be simulated, while the temperature inside the pulp chamber was initially stabilized at an average value close to the body core temperature (approximately 37°C)^6^ ^,^ ^8^ ^,^ ^12^ or lower.^10^ ^,^ ^11^ Conversely, other studies focused only on measuring temperature changes during exposure to LCUs, without simulating tooth physiological conditions.^9^ ^,^ ^13^ Because of differences between these approaches, along with variance in LCU types, radiant emittances, and characteristics of teeth,^4^ ^,^ ^9^ ^,^ ^11^ ^,^ ^13^ ^–^ ^18^ a wide range in temperature value increases inside the pulp chamber, ranging from 1.5 to 23.2°C, is found in the literature.^4^ ^,^ ^9^ ^,^ ^11^ ^,^ ^13^ ^–^ ^18^ However, despite such differences in results and methodologies, many *in vitro* studies concluded that the use of some LED LCUs can cause an increase in temperature values within the pulp chamber higher than the threshold temperature increase considered harmful for the pulp (5.5°C).^5^

Despite the important impact of these conclusions based on *in vitro* methods on the attention of researchers and manufacturers to this possible issue, it is reasonable to assume that *in vitro* conditions do not fully reproduce the complex physiological mechanism involved in the real *in vivo* condition. As a consequence, *in vitro* analysis is expected not to be capable of precisely reproducing *in vivo* PT when intact teeth are exposed to a LED light using varying exposure modes. However, due to the lack of *in vivo* studies that evaluated PT changes during heat stimulus when most *in vitro* studies were published in the past, and because of differences between tested teeth and tooth condition among studies, no contemporary data are available to confirm how well an *in vitro* model can reproduce temperature changes seen in the *in vivo* model, when under thermal stimuli such as the exposure to light emitted from a powerful LED LCU. Recently, an *in vivo* methodology was published that measured PT within the pulp tissue of human premolars.^19^ ^–^ ^21^ In that approach, the temperature probe of a wireless temperature acquisition system, previously calibrated using National Institute of Standards and Technology (NIST-traceable) methods, is inserted within the human pulp tissue through an occlusal access, and real-time PT is monitored during thermal stimuli.

Thus, the purpose of this study was to evaluate how similar an *in vitro* model is able to reproduce temperature increase (ΔTEMP) values compared to the *in vivo* model, in anesthetized intact, unrestored, human upper premolars, in order to validate the *in vitro* methodology. The unique feature of this work was that the same premolar teeth tested for *in vivo* temperature rise were extracted for orthodontic treatment, and were subsequently tested in a clinically relevant *in vitro* system. In addition, the same LCU used in the *in vivo* analysis was also used for *in vitro* analysis. The tested alternative hypotheses were that (1) there are no significant differences in ΔTEMP values and time for temperature to reach maximum (ΔTIME) measurements between *in vitro* and *in vivo* models, and (2) both the *in vivo* and *in vitro* models show a direct, positive correlation between applied radiant exposure to intact facial tooth surface and both ΔTEMP and ΔTIME.

## Methodology

### *In vivo* measurement of pulp temperature increase

After approval by the local Ethics Committee (protocol #255,945), study participants requiring extraction of upper right and left first premolars for orthodontic reasons were selected from the Orthodontic specialization program in Ponta Grossa, Brazil, and were recruited in February, 2013. The participants were seen between March and April, 2013. Inclusion and exclusion criteria were based on previous *in vivo* studies,^19^ ^–^ ^21^ and included (1) treatment plans indicating premolar extractions for orthodontic reasons, (2) the presence of healthy, intact, non-carious, and non-restored, fully erupted treatment teeth, and (3) patients with well-controlled health conditions that allowed all procedures involved in the research to be performed with minimal risk. Exclusion criteria included (1) patients who did not agree to volunteer for the study, (2) patients not meeting all of the inclusion criteria.

The *in vivo* real-time temperature analysis within the pulp was evaluated following a method previously described in the literature.^19^ ^–^ ^21^ A single tooth at a time received approximately 1.8 ml of 2% mepivacaine hydrochloride (36 mg) with 1:100,000 epinephrine (18 µg) (Mepiadre, DFL Industria e Comércio, Rio de Janeiro, RJ, Brazil) by infiltrative and intraligamental anesthesia. The tooth was isolated using rubber dam, and a small preparation was made in the center of the occlusal surface, using a round diamond bur (#1015, KG Sorensen, Cotia, São Paulo, Brazil) in a high speed handpiece, under air-water spray, until the preparation pulpal floor was near the buccal pulp horn. Then, minute pulp exposure was obtained using a diamond bur (2134, KG Sorensen), with no pulp bleeding. Two calibrated T-type temperature probes were connected to a wireless, NIST-traceable, temperature acquisition system (Temperature Data Acquisition - Thermes WFI, Physitemp, Clifton, NJ, USA) and were immersed in a room temperature (approximately 22.0°C), 0.9% sterile saline solution. Both thermocouples indicated similar temperature values. After pulp exposure was obtained, one probe was inserted directly into the pulp chamber through the narrow access created occlusally, and was positioned over a small groove created on the buccal cusp tip ridge to remain stable, while PT was measured, and ensure that the 1-cm long probe tip penetrated approximately 4 mm into the pulp chamber. The other probe was kept in saline solution and acted as internal reference, confirming that any PT change could be attributed exclusively to exposure from the curing light. The room temperature probe reading remained stable, as the ambient air temperature was controlled by air conditioning set to approximately 22°C. The occlusal preparation was filled with provisional restorative material (Cavitec, CaiTHEC Ltda, São José dos Pinhais, PR, Brazil) to minimize heat loss from the tooth through the preparation walls and pulp access, while the probe remained in place. PT reached a stable baseline value (approximately 35°C)^20^ after approximately 15 min of real-time analysis, during which data were continuously acquired every 0.2 s. The LCU tip was positioned against and as close as possible to the buccal tooth surface and the tooth was sequentially exposed to the radiant output from a Polywave^®^ LED LCU (Bluephase 20i, Ivoclar Vivadent, Schaan, Principality of Liechtenstein) using the following exposure modes (EMs): 10-s at low intensity (10-s/L); 10-s at high intensity (10-s/H); 5-s at Turbo intensity (5-s/T); and 60-s at high intensity (60-s/H). These exposure modes were selected because they are the most clinically relevant modes used for a wide variety of clinical applications. A 7-min time span between each exposure was allowed for the PT to return to baseline levels. The sequence of EMs was randomly determined and the operator was not aware of which mode was being used. The time of data acquisition when each light mode was applied was recorded using a digital time counter that started recording simultaneously to the beginning of real-time PT analysis, so that time of light activation and correlated temperature measurement could be precisely made. The probe was removed from the tooth at the end of the temperature data acquisition, and the tooth was extracted as planned. Radiographs were taken from the proximal side of the extracted tooth with the probe in position as it was intraorally in order to confirm the proper insertion depth and location of the probe within the pulp chamber during temperature measurement.

A laboratory grade spectroradiometer (USB 2000+, Ocean Optics, Dunedin, FL, USA) connected to a 6-in integrating sphere (Labsphere, North Sutton, NH, USA), previously calibrated using a NIST-traceable light source was used to evaluate the spectral power of the tested EMs. In this regard, the LCU tip end was positioned at the entrance of the integrating sphere, so that all light emitted from the unit was captured. Wavelength-based, spectral and power emission during each EM were recorded using software (SpectraSuite v2.0.146, Ocean Optics) between 350 to 550 nm, which also provided the total emitted power value for that wavelength range. Radiant emittance values of each EM (mW/cm^2^) were determined as the total measured power value was divided by the light-emitting area of LCU distal tip end. That analysis was performed before the beginning of both *in vivo* and *in vitro* analyses. This value was then multiplied by the light exposure duration in order to derive the value of radiant exposure applied to each tooth surface for each light output mode (J/cm^2^). The corresponding radiant exposure obtained for each EM was as follows: 10-s/L: 6.56 J/cm^2^; 10-s/H: 12.44 J/cm^2^; 5-s/T: 11.02 J/cm^2^; 60-s/H: 74.64 J/cm^2^.

### *In vitro* analysis of PT increase

**Figure 1:**
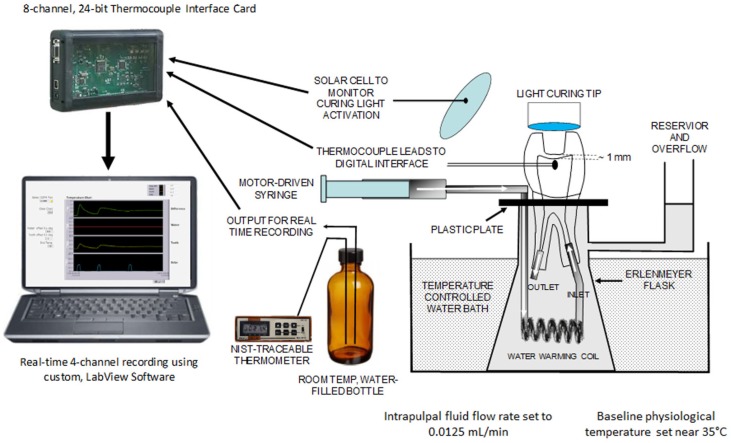
Diagram of *in vitro* test assembly

The same premolars and LCU used in the *in vivo* study were tested in the *in vitro*, so any possible difference between outcomes would be exclusively attributed to the differences between the two models. The extracted teeth were stored in 0.1% thymol (Symrise GmbH, Holzminden, Germany) until the moment they were fixtured to and tested in the *in vitro* model previously established.^10^ In that approach, a test assembly simulated the *in vivo* environment: controlled intrapulpal physiologic baseline temperature of approximately 35°C^19^ and a simulated intrapulpal fluid flow. Figure 1 displays the components of the test setup. A K-type thermocouple (part #TT-K-30-SLE, Omega Engineering Inc., Stamford, CT, USA) was fabricated by joining wire ends with a spot-welder (Model R660, Rocky Mountain/Orthodontics, Denver, CO, USA). Apical portions of the root canals were enlarged and the pulp tissue was removed from the pulp chamber through the enlarged root canals using barbed broaches of various sizes. The thermocouple wire was placed into the pulp chamber through the same occlusal access opening made on the teeth during the *in vivo* analysis. The thermocouple junction was placed in a similar position to that of the thermocouple used in the *in vivo* analysis. In order to assure similar placement, x-ray analyses were used to compare thermocouple positioning for each tooth between the *in vivo* and *in vitro* conditions. Occlusal access was sealed and stabilized using acid etching and a flowable composite (Aeliteflo, Lot Number 1200001055; Bisco Inc., Schaumburg, IL, USA). Small sections of 16-gage stainless steel tubing were attached to the root ends using acid etching using the same flowable composite. A section of flexible plastic tubing was connected to one tube end and a portion of the remaining end was coiled to increase surface area in contact with the warmed water in the Erlenmeyer flask in which it was immersed. The tubing continued through a hole in the plastic plate placed over the water-filled Erlenmeyer flask (Figure 1). The distal end of the tubing was connected to a water-filled, 20 mL glass syringe. The syringe body was held in a fixed position, while the plunger end was connected to a screw-driven extension of an infusion pump (Model 600-900, Multispeed Transmission Pump, Harvard Apparatus Company, Dover, MA, USA). The rate at which water was circulated in the tooth was calculated from existing literature. The average volume of pulp chamber in a human maxillary first premolar is approximately 18.2 mm^3^ (0.0182 cc).^22^ Assuming that the density of human pulp is similar to that of connective tissue (1.027 g/cc),^23^ the tissue mass in this tooth would be approximately 0.01869 g. The reported pulpal flow rate in dogs is 33.32 ml/min for each 100 g of tissue.^24^ Applying this rate to the mass of pulpal tissue calculated in a maxillary upper first premolar yields a flow rate of 0.0062 ml/min (6.2 μl/min). The setting used on the infusion pump closest to this value, for the syringe size used (20 ml), was 0.0125 ml/min (12.5 μl/min), which falls well within values others have used for similar test setups.^11^ The roots of the prepared tooth were placed through an opening in the plastic plate that covered the top of the Erlenmeyer flask. The peripheral area remaining between the coronal root surface and the opening of the plastic plate was sealed using the same flowable composite. The flask itself rested in the water of a temperature–controlled bath (Model 1-2000, Thermo-Lift, Bruchler Instruments Inc., Fort Lee, NJ, USA). Water temperature was thermostatically controlled to provide an intrapulpal temperature of approximately 35.5°C (±0.5°C), which is similar to that of the *in vivo* condition.^20^

The thermocouple output was connected to a multi-channel thermocouple interface card (TCIC, Omega Engineering), where it was electronically cold-junction compensated, digitized, and sent to software (LabView, v 7.1, National Instruments, Austin, TX, USA) on a personal computer. The software both visually and digitally recorded the thermocouple temperature in real-time, at a rate of 10 data points *per* second. A photocell was placed in close proximity to the emitting end of the light curing unit in order to generate voltage when the light was activated. The output of the photocell was included with those of the other data feeds. In this manner, it was possible to determine the exact time when the light unit was activated and deactivated, being correlated with changes in intrapulpal temperature. Lastly, a similar type thermocouple lead was placed into a large, capped bottle of water maintained at room temperature. The output from this lead was registered in real-time along with that of the photo cell and intrapulpal thermocouple. At baseline, a temperature offset of the water bottle value was adjusted in order to provide a zero-difference temperature value between that value and the real-time temperature measured within the pulp. Thus, an additional digital recording channel was created that allowed a temperature rise above the observed intrapulpal value to be registered from a zero-degree level, in real-time. A separate, similar K-type thermocouple was placed into the room-temperature water bottle, and its output was fed into a dedicated, NIST-traceable digital temperature measurement device (model AN6503, Analogic Corporation, Danvers, MA, USA). In this manner, the temperature calibration of the main *in vitro* temperature measurement system could be achieved by adjusting the water offset temperature to match that of the NIST-traceable device. The real-time profile data of *in vitro* and *in vivo* PT increase were plotted into line graphs (Excel 2007, Microsoft), which were used to determine ΔTEMP and ΔTIME.

Time constant (τ) of each thermocouple was determined as previously described.^19^ For the temperature data acquisition system used in the *in vivo* model, the τ obtained was 1.46 s, while a τ of 0.05 s was observed for the thermocouple used in the *in vitro* model. In other words, the time required for the temperature data acquisition system to provide a 1-degree Centigrade temperature change using the *in vivo* PT analysis was approximately 0.07 s, while 0.0029 s were required for the thermocouple to provide the same 1-degree Centigrade temperature change using the *in vitro* setup.

### Statistical analyses

The ΔTEMP and ΔTIME data obtained *in vivo* and *in vitro* were subjected to two-way repeated measures ANOVA, followed by Bonferroni's *post-hoc* test at a pre-set alpha of 0.05. Linear regression analysis was performed to examine the relationship between applied radiant exposure level and ΔTEMP, as well as between radiant exposure and ΔTIME in both conditions. The comparison between the slopes of both regression lines obtained from the *in vivo* and *in vitro* data was performed by performing t-tests as well as by comparing possible overlaps in the 95% confidence intervals (CI) for the mean slope values. No overlap indicated a significant difference, and overlap suggested no significant difference. Post-hoc power analysis was performed for the statistical analysis of ΔTEMP and ΔTIME. All analyses were performed using statistical software on a personal computer (Statistics 19, SPSS Inc, IBM Company, Armonk, NY, USA).

## Results

### *In vivo* and *in vitro* ΔTEMP and ΔTIME during curing light exposures

**Figure 2:**
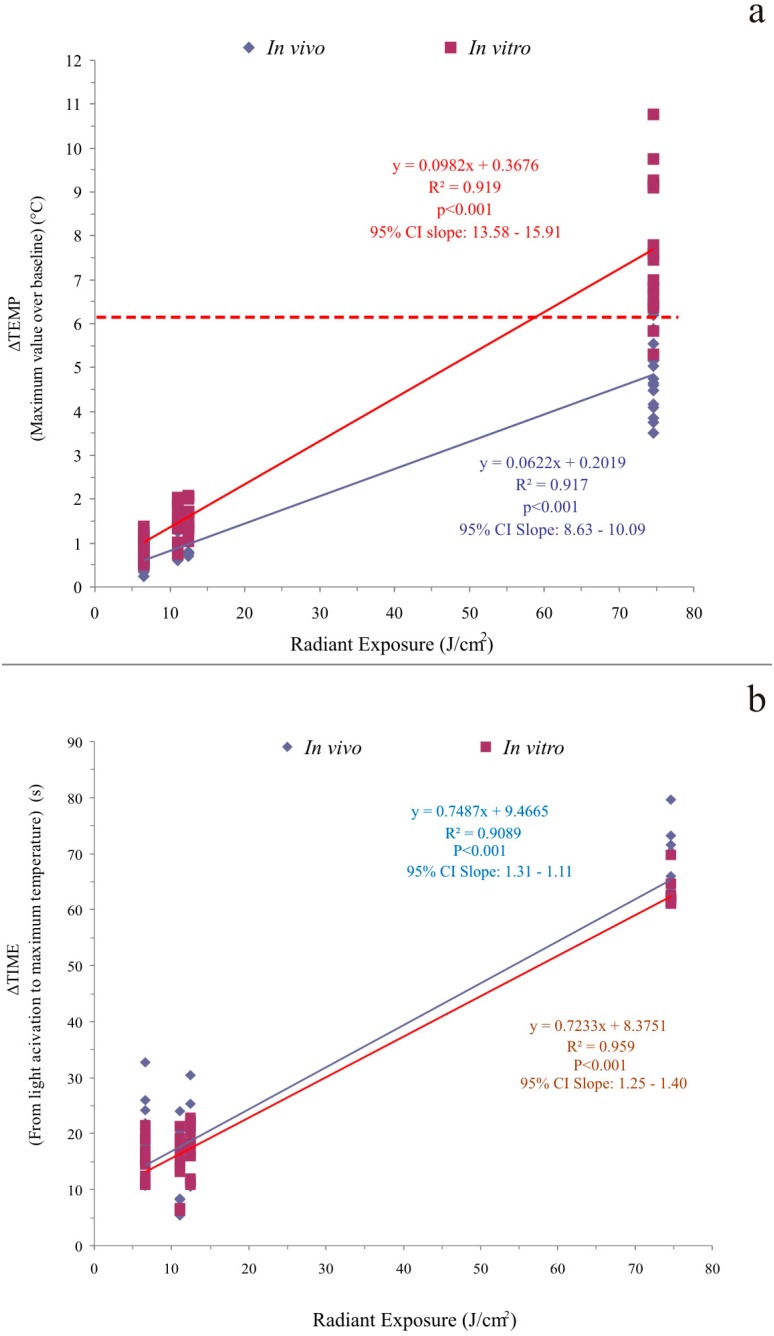
Regression analysis plot of *in vivo* and *in vitro* (a) ΔTEMP above pre-exposure, baseline temperature (˚C) within the pulp chamber vs. applied radiant radiant exposure, and (b) ΔTIME (b)

For the number of evaluated teeth (n=15), the *in vivo* study was adequately powered for EM and condition (*in vitro* and *in vivo)* factors (over 99,0%; α=0.05). Table 1 presents the comparison between *in vitro* and *in vivo* ΔTEMP values. Overall, the *in vitro* model recorded significantly higher ΔTEMP values than the *in vivo* model, regardless of EM. Although a significant, positive relationship [*in vivo:* (r^2^=0.917; *p*<0.001; *in vitro:* r^2^=0.919; *p*<0.001)] was observed between delivered radiant exposure and ΔTEMP in both models, the slope of the regression line created *in vitro* was significantly higher than that observed *in vivo* (*p*<0.001, no overlap in 95% CI of slope values; Figure 2a). As a result, for the 10-s/H, 5-s/T, and 60-s/H EMs, the *in vitro* ΔTEMP values were approximately 1.6 times higher than *in vivo* values, while *in vitro* ΔTEMP values were approximately ١.٨ times higher than *in vivo* results when the 10-s/L EM was used. However, these differences were only 0.4°C for 10s-L, and 0.6°C for both the 10s-H and 5s-T EMs. Using the 60s-H setting produced 2.9˚C greater value in the *in vitro* model than when testing *in vivo*.

**Table 1:** Mean ΔTEMP (SD) (°C) of *in vivo* and *in vitro* results values during exposure using different exposure modes

	Exposure duration - Curing Mode			
Test Condition	10s-L	10s-H	5s-T	60s-H
*In vivo*	0.5 (0.2)^Bc^	1.0 (0.3)^Bb^	1.0 (0.3)^Bb^	4.8 (1.0)^Ba^
*In vitro*	0.9 (0.3)^Ac^	1.6 (0.3)^Ab^	1.6 (0.4)^Ab^	7.7 (1.6)^Aa^

Means followed by similar letter (uppercase letters: within column (between test conditions within an EM); lower case letters: within row (within test condition among EMs) are not significantly different. L = low H = High T = Turbo n = 15 Specimens per condition

Use of the 60-s/H EM caused the highest ΔTEMP in both *in vivo* and *in vitro* models (*p*<0.001), whereas the 10-s/L EM produced the lowest values (*p*<0.001). Despite the *in vivo* ΔTEMP average values being lower than 5.5°C, some teeth exhibited higher ΔTEMP values than that threshold temperature (Table 1). On the other hand, the *in vitro* model indicated that, when the same 60-s/H EM was used, all teeth produced ΔTEMP values exceeding 5.5°C. In both *in vivo* and *in vitro* models, ΔTEMP values of 10-s/H group were not significantly different from those of 5-s/T EMs, which in turn, produced significantly higher ΔTEMP values than did the 10-s/L mode (*p*<0.001).

Despite such differences in ΔTEMP values, no significant differences in ΔTIME values were noted when the *in vivo* model was compared to that of *in vivo* results, regardless of EM (Table 2). In this regard, for both conditions, the 5-s/T mode generated the shortest ΔTIME, while 60-s/H EM produced to the highest intervals. The 10-s/H and 10-s/L EMs developed higher ΔTIME values than did the 5-s/T mode, and lower values than the 60s-H mode. A significant, similar, positive relationship (*in vivo:* r^2^=0.917; *p*<0.001; *in vitro:* r^2^=0.919; *p*<0.001) was also observed between delivered radiant exposure and ΔTIME for both test conditions (Figure 2b). No significant difference was noted between the slopes of the regression lines from *in vivo* and *in vitro* data (overlap in the 95% CI of mean slope values).

**Table 2:** Mean time (ΔTIME) (SD) (s) from start of exposure to reach maximum intrapulpal temperature (ΔTEMP) for *in vivo* and *in vitro* test conditions, using different exposure durations and exposure modes

	Exposure duration - Curing Mode			
Test Condition	10s-L	10s-H	5s-T	60s-H
*In vivo*	18.9 (6.1)^Ab^	18.2 (5.9)^Ab^	13.5 (6.8)^Ac^	65.7 (5.5)^Aa^
*In vitro*	15.9 (3.4)^Ab^	17.2 (4.2)^Ab^	13.6 (5.0)^Ac^	62.6 (2.2)^Aa^

Means followed by similar letters (uppercase letters: within column (between test conditions within an EM); lower case letters: within row (within test condition among EMs) are not significantly different. L = low H = High T = Turbo n = 15 Specimens per condition

**Figure 3:**
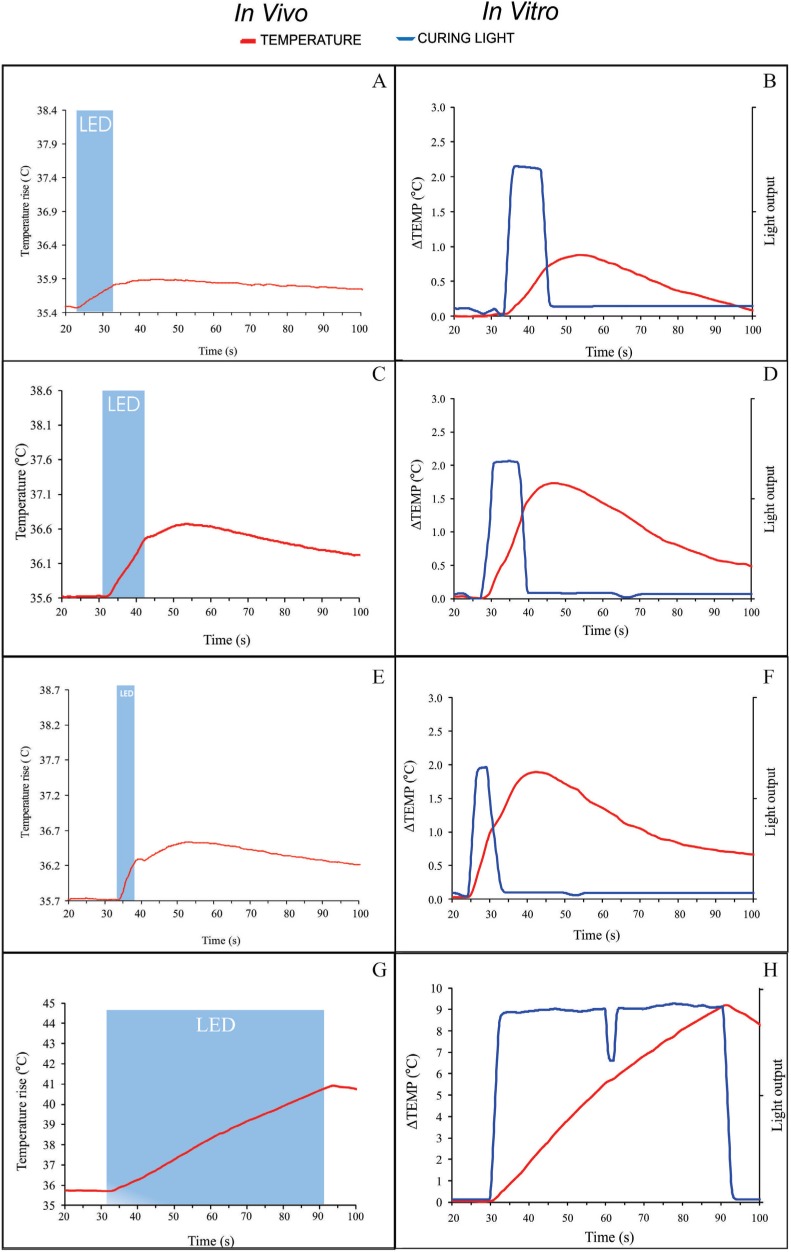
Examples of *in vivo* and *in vitro* real time temperature increase in the pulp chamber during exposure to light using 10-s/L (a and b), 10s/H (c and d), 5-s/T (e and f), and 60-s/H (g and h) EMs. The blue area represents the time interval when teeth were exposed to the curing light

For both *in vivo* and *in vitro* models, the time/temperature profiles (Figure 3) of PT increase during exposure to the LED LCU showed a rapid increase in PT rise approximately 2 s after light activation, while higher radiant exposure levels caused greater magnitude of the PT increase. For all EMs, the *in vitro* PT increase profile was also more pronounced during and after exposure to LED light than that observed *in vivo.* For most EMs, when the LCU shut off, PT continued to increase for a few seconds for both test conditions, except for the 60-s/H mode, in which PT values dropped immediately after the LED light shut off (Figures 3g and 3h). In teeth exposed to the 5-s/T EM *in vitro*, approximately half of the total PT increase occurred during exposure to the LED light, while the other half was noted after the light shut off (Figure 3f).

## Discussion

To the extent of our knowledge, this is the first study comparing *in vitro* results of temperature rise within the pulp chamber during exposure to light emitted from a Polywave LCU with those obtained *in vivo* in intact, human premolars, in an attempt to validate the *in vitro* model. Based on the current findings, the *in vitro* model recorded higher ΔTEMP values than the *in vivo* model, regardless of EM. It is worth noticing, however, that the greatest difference in ΔTEMP between *in vitro* and *in vivo* models was only noted when 60-s/H was delivered, whereas only small differences in ΔTEMP were observed when other, more clinically relevant, EMs were delivered to the teeth. Curiously, despite such differences, no significant difference in ΔTIME was observed between both test conditions, even when *in vitro* ΔTEMP was 1.6 times higher than *in vivo* values. Such a finding infers that the *in vitro* rate of PT increase was higher than that observed in the *in vivo* model, as illustrated by the time/temperature profiles of PT increase (Figure 3). Therefore, the first alternative hypothesis, stating that there are no significant differences in ΔTEMP values and ΔTIME measurements between *in vitro* and *in vivo* models, was partially rejected. Because the LED LCU, EMs and teeth were the same for both test conditions, such divergence between these results may be attributed to the dynamic regulatory mechanism of pulp tissue for heat distribution during temperature changes in this tissue used to dissipate heat transferred by external thermal stimuli throughout the dentine/pulp complex.^6^ ^,^ ^25^ ^,^ ^26^ In other words, when any external thermal stimuli generates more heat, fluid movement, either inwards or outwards from the pulp, will increase in an attempt to reduce the magnitude of PT rise.^6^ ^,^ ^25^ ^,^ ^26^ For this reason, the actual *in vivo* pulp regulatory system has shown to be more effective in dissipating external heat than the simulated pulpal fluid flow in the *in vitro* model.

The importance of the increase in pulp blood flow rate during exposure to an LED light may also be confirmed by the comparison between *in vivo* and *in vitro* linear regression analyses. Although both *in vivo* and *in vitro* models presented significant, positive relationships (*in vivo:* r^2^=0.917; *p*<0.001; *in vitro:* r^2^=0.919; *p*<0.001) between delivered radiant exposure and ΔTEMP, the significantly lower regression slope obtained from the *in vivo* results (Figure 2a) infers that an *in vivo* defense mechanism of pulp blood flow against heat rise became more effective as higher radiant exposure values were delivered to teeth. Therefore, the resulting lack of parallelism between both regression lines for ΔTEMP implies that both models presented a significant, positive, but not similar relationship between ΔTEMP and radiant exposure. For this reason, the second hypothesis was partially rejected. One could state that exposure modes delivering radiant exposure values between 20 and 70 J/cm^2^ should be added to the sequence of EMs used in the current study, so a more reliable regression analysis could be obtained. However, a recent *in vivo* study evaluating PT increase in human premolars with Class V preparations observed similar relationship between radiant exposure values and PT rise.^21^ In that study, another EM delivering a radiant exposure of approximately 37.3 J/cm^2^ was tested. Therefore, it is reasonable to expect that the regression analysis using the current data provides a reliable relationship between radiant exposure values and PT increase.

Another reason for such differences in ΔTEMP and rate of PT increase between *in vitro* and *in vivo* models is the difference between the content inside the *in vitro* pulp chamber, and that inside the *in vivo* pulp chamber, once the pulp tissue was removed from the pulp chambers before the extracted teeth were used in the *in vitro* model. In this regard, when the enamel surface is hit by blue light, part of the light energy is either reflected or converted into thermal energy, while the remaining portion passes through to the substrates below.^27^ Because the thermal conductivity and thermal diffusivity of pulp tissue and blood (0.63) are close to those of water (0.58),^28^ ^,^ ^29^ the converted thermal energy released by the inner dentin passes through pulp tissue, blood, or water to reach the thermocouples similarly. On the other hand, the remaining portion of light that passes through enamel and dentin interacts differently between the *in vitro* and *in vivo* environments inside the pulp chamber. In the *in vivo* model, due to blood content, pulp is rich in hemoglobin, a chromophore with a coefficient of absorption within the blue light emission wavelength range,^30^ so when blue light reaches the pulp tissue, photons are strongly absorbed by blood chromophores to be partially converted into thermal energy,^31^ resulting in a slower PT increase *in vivo* than that observed *in vitro.* Furthermore, in this context, because of the constant blood flow, the warmed chromophores from absorbed photons are quickly replaced by cooler ones, so most of the heat generated by irradiance of this tissue is dissipated. However, it should be noted that the intensity of the curing light is severely attenuated by the thick buccal wall of intact premolars,^32^ ^,^ ^33^ so the influence of residual irradiance on PT rise should be lower than the influence of thermal conduction from the heated dentin substrate. Only further investigation could confirm such an assumption.

In the current study, although the *in vitro* ΔTEMP values were significantly higher than those observed *in vivo*, both conditions had no influence on the EM effects on PT rise. This fact seems valid because both *in vivo* and *in vitro* 10-s/L exposures showed the lowest PT values, which were significantly lower than when the 10-s/H and 5s-T modes were used, while the 60-s/H mode produced the highest ΔTEMP. Indeed, because of the aforementioned differences between test methods, the *in vitro* 60-s/H EM caused higher ΔTEMP (7.7±1.6°C) than the well-known threshold temperature of 5.5°C, in all extracted teeth, while the same EM applied *in vivo* resulted in lower average increase (4.8±1.0°C) than that threshold temperature.^5^ Based on these findings, it is evident that, overall, *in vitro* studies may overestimate the effects of LCU exposure on pulp chamber temperature increase when high radiant exposure values are delivered to the teeth.

Although much of the differences in PT rise between *in vivo* and *in vitro* methodologies may be attributed to the physiological response of the pulp tissue against heat rise, some features in these methodologies may have contributed to the differences in the temperature rise within the pulp chamber. For instance, the Class I preparation in the *in vivo* method was sealed with provisional restorative material, while the Class I preparation in the *in vitro* methodology was sealed with flow resin composite to keep the simulated fluid flow within the pulp chamber. Because zinc-based cements, such as the provisional material used in the i*n vivo* model, allow for greater heat transfer than do resin composites,^34^ these provisional restorative materials may have allowed more heat to be released through the occlusal cavity than when the Class I preparation was sealed with resin composite.

Despite these differences, the current results demonstrated that the *in vitro* model is capable of detecting temperature changes as the delivered radiant exposure values increased in a similar pattern as that observed in the *in vivo* model. Therefore, the only flaw observed in the *in vitro* model is the overestimation of the temperature rise within the pulp chamber. In this regard, some studies have shown that the increase in the *in vitro* simulated fluid flow rate resulted in lower temperature rise within the pulp chamber.^6^ ^,^ ^11^ ^,^ ^35^ Based on this evidence, increasing fluid flow rate in the *in vitro* model could be a valuable alternative to compensate for such difference between *in vivo* and *in vitro* models. Further studies are required to determine the settings of *in vitro* methodologies to provide temperature increase values within the pulp chamber similar to those observed *in vivo.*

## Conclusions

Within the limitations imposed by the methodologies used, the following conclusions may be made:

1. The *in vitro* model detected higher PT increase than the *in vivo* model, when the same teeth were exposed to the same exposure modes from the same Polywave^®^ LED LCU;
2. *In vitro* PT increase values were close to *in vivo values* when clinically relevant exposure modes were delivered (between 7 and 12 J/cm^2^ for 10- and 5-s exposures), and
3. A significant, positive and non-parallel correlation was observed between delivered radiant exposure and PT increase for both *in vivo* and *in vitro* models, so the influence of varying exposure modes on PT increase was similar for both models.
